# Radiologists’ leading position in image-guided therapy

**DOI:** 10.1007/s13244-012-0213-9

**Published:** 2013-01-17

**Authors:** Thomas Helmberger, Luis Martí-Bonmatí, Philippe Pereira, Alice Gillams, Jose Martínez, Johannes Lammer, Katarina Malagari, Afshin Gangi, Thierry de Baere, E. Jane Adam, Coen Rasch, Volker Budach, Jim A. Reekers

**Affiliations:** 1 Department of Diagnostic and Interventional Radiology, Neuroradiology and Nuclear Medicine, Klinikum Bogenhausen, Munich, Germany; 2 Radiology Department, La Fe University and Polytechnic Hospital, Valencia, Spain; 3 Radiology, Department of Medicine, Universidad de Valencia, Valencia, Spain; 4 Department of Radiology, Minimally Invasive Therapies and Nuclear Medicine, SLK-Kliniken GmbH, Heilbronn, Germany; 5 Radiology, The London Clinic, London, United Kingdom; 6 Hospital Universitario La Fe, Radiología Vascular Intervencionista, Valencia, Spain; 7 Department of Angiography and Interventional Radiology, University Vienna, AKH, Vienna, Austria; 8 Second Department of Radiology, University of Athens, Attikon Hospital, Athens, Greece; 9 Interventional Radiology, Strasbourg Hospital, Strasbourg, France; 10 Institut de Cancérologie Gustave Roussy, Villejuif, France; 11 Department of Radiology, St. George’s Hospital, London, United Kingdom; 12 Radiation Oncology, The Netherlands Cancer Institute, Amsterdam, The Netherlands; 13 Department for Radiation Oncology, Campus-Mitte and Campus-Virchow, Charité University Medicine, Berlin, Germany; 14 Department of Radiology, Academic Medical Center, University of Amsterdam, Amsterdam, The Netherlands

**Keywords:** Image guided therapy, Health care, Patient service, Quality standards

## Abstract

Image-guided diagnostic and therapeutic procedures are related to, or performed under, some kind of imaging. Such imaging may be direct inspection (as in open surgery) or indirect inspection as in endoscopy or laparoscopy. Common to all these techniques is the transformation of optical and visible information to a monitor or the eye of the operator. Image-guided therapy (IGT) differs by using processed imaging data acquired before, during and after a wide range of different imaging techniques. This means that the planning, performing and monitoring, as well as the control of the therapeutic procedure, are based and dependent on the “virtual reality” provided by imaging investigations. Since most of such imaging involves radiology in the broadest sense, there is a need to characterise IGT in more detail. In this paper, the technical, medico-legal and medico-political issues will be discussed. The focus will be put on state-of-the-art imaging, technical developments, methodological and legal requisites concerning radiation protection and licensing, speciality-specific limitations and crossing specialty borders, definition of technical and quality standards, and finally to the issue of awareness of IGT within the medical and public community. The specialty-specific knowledge should confer radiologists with a significant role in the overall responsibility for the imaging-related processes in various non-radiological specialties. These processes may encompass purchase, servicing, quality management, radiation protection and documentation, also taking responsibility for the definition and compliance with the legal requirements regarding all radiological imaging performed by non-radiologists.

## Introduction

During the last 30 years, image-guided therapies—with guidance ranging from ultrasound (US) to magnetic resonance imaging (MRI)—have been used increasingly to treat patients suffering from oncological and vascular diseases. Parallel to the development of endovascular and intratumoral techniques, advancements in imaging have contributed greatly to improve the clinical results of percutaneous minimally invasive treatments in nearly all medical fields and specialties.

Image-guided therapy (IGT) can be defined as any type of local therapy based on identifying a target, targeting the target, monitoring the procedure, and often monitoring the local effect (immediate follow-up) by any kind of radiological imaging at various contrast resolutions, as well as spatial and temporal resolutions for active localisation and supervision. These procedures are performed in environments providing the necessary imaging techniques, such as radiology, nuclear medicine, radiotherapy and surgical suites.

Compared with conventional surgical approaches, IGT uses technically generated and processed imaging data from any imaging equipment acquired before, during and after a specific procedure. This means that planning, performing and monitoring, as well as controlling a procedure is based and dependent on the “virtual” reality provided by imaging.

In this white paper, IGT will be reviewed with regard to:

- Defining state-of-the-art techniques focused on diagnosis and therapy, including present and future developments.
- Technical, methodological and legal (radiation protection, “licensing” according to the assignment to medical specialties) characteristics of imaging guidance and therapy.
- Medical specialty-specific limitations and “borders” for present and future techniques (e.g. hybrid operating rooms).
- A framework for increasing the profile and recognition of IGT in terms of training and education, research and public awareness.
- The role of radiologists in defining quality standards and professional relationships within IGT initiatives outside radiology departments.

## Role of IGT at present and future developments

The role of image guidance for percutaneous and intravascular treatments is to ensure a precise, effective and safe therapy (e.g. complete tumour destruction without injury to critical structures). Therefore, image guidance during interventional therapies may provide an exact planning and targeting, rapid imaging for positioning of the instrument, reliable monitoring during the course of treatment, and control during the same session [1]. Planning before or during the treatment means exact localisation of the targeted tissue and surrounding organs; targeting corresponds to the placement of a catheter or probe into the targeted area; monitoring describes the observation of changes during therapy; control is related to imaging of the response to treatment and efficacy, and early diagnosis of adverse effects during the session.

Therefore, ideal qualities of imaging for guidance of interventional therapies include:

- Adequate target/tissue contrast with clear demarcation between targeted structure and the surrounding anatomy.
- Ultrafast real-time or near real-time imaging.
- Multiplanar and interactive imaging capabilities.
- Visualisation of therapy effects, including safety margin, for ablation therapy during and at the end of the procedure, as far as possible including three-dimensional (3D) evaluation.

Imaging requirements are often different depending on the kind of intervention, principally between percutaneous, vascular and open interventions. Nowadays, no single imaging investigation can encompass the entire clinical requirements for minimally invasive therapy. For example, MRI—due to superior sensitivity and specificity even in small lesions—might provide image guidance in percutaneous ablative therapy, but fast, near real-time imaging, possible with US and MRI, can also easily facilitate percutaneous access. However, it is also possible to have fast and accurate guidance by CT, applying CT fluoroscopy imaging, with the drawback of causing radiation exposure for both the interventionalist and for the patient.

For intravascular therapies, an exact knowledge of the vascular anatomy (for access routes) and associated abnormalities (stenosis, occlusion, tumour feeding vessels) is essential for planning the treatment and for proper catheter positioning that usually may be done by 2D digital subtraction angiography. Recent advances in imaging technology produce CT-like soft-tissue images, allowing identification of complex vascular situations, which are provided in well-equipped environments. Nevertheless, it is debatable whether such techniques have to be implemented in all hybrid operating theatres with respect to the limited access, limited need and the related high costs. In this context, treatment and cost effectiveness should overcome particular professional interests of specific specialties.

Intraoperative open surgery also clearly benefits from IGT, as the precise location and definition of the characteristics of the lesion cannot be possible without the use of virtual visualisation and parametric demonstration of tissue changes. As an example, CT and MRI guidance are increasingly used during brain surgery in order to locate the pathological abnormality. IGT has revolutionised traditional surgical techniques by providing surgeons with a way to navigate through the body using several 3D images and tools. A future approach to further improve the precision of targeting and guiding in IGT may be an integration of robotic assistance into interventional suites. Compatible robotic assistance should operate without interfering with instrument placement, during control after positioning and at controlling after treatment.

Nevertheless, the role of image guidance for a growing wide variety of medical specialities (e.g. surgery, orthopaedic surgery, neurosurgery, cardiology and cardiac surgery) and procedures, not primarily related to radiology, is permanently growing. This role is mainly driven by the sophisticated opportunities offered by medical computing and radiological image guidance with regard to precision and minimal invasiveness [2]. However, the impact of radiology on the regulatory medico-legal, technical and radioprotection issues in this field have not yet been defined. Since an increasing number of procedures will probably be performed by non-radiologists, several main questions have to be addressed:

- How should the radiology training requirements for non-radiologists be provided?
- How should the technical and radioprotection related responsibilities for radiological imaging systems used by non-radiologists be organised?
- How should radiologists be involved in the practical routine use of non-radiological image-guided procedures in clinical practice?

Considering the almost pan-European medical reality with decreasing staff resources and increasing diversification and subspecialisation, radiologists have to stress the fact that within a cooperative, goal-oriented and multidisciplinary environment, the specialty-specific knowledge should confer upon radiologists a significant impact on the overall responsibility for all imaging-related processes in various non-radiological specialties (such as purchase, servicing, quality management, radiation protection and documentation). Furthermore, radiologists should take responsibility for the definition and compliance with the legal requirements regarding all radiological imaging, especially if non-radiologists have to be trained in the use of imaging technology for guidance of therapy.

## Classification of IGT and IGT Catalogue

IGT can be classified according to the type of procedure, the target organ/type of tumour and the intended result. An important fact is that a reasonable number of these procedures are performed by non-radiologists. As long as radiation exposure is a component of most image guidance (e.g. intraoperative fluoroscopy in hybrid operating theatres), national and international radiation protection guidelines have to be fulfilled and the referring, practicing, and operating part under Euratom legislation have to be qualified and certified.

Most IGT procedures relate to diagnostic and therapeutic interventions such as evacuation/decompression, revascularisation/devascularisation, pain relief, critical life conditions (e.g. acute bleeding) and tumour control (palliative, curative).

The catalogue of IGT procedures is impossible to summarise and continuously enlarges. For clarification purposes, the following is a schematic approach:

- ***IGT in non-tumour conditions such as:***

1. Transvascular and endovascular procedures
  - Recanalisation, revascularisation by percutaneous balloon angioplasty, stent placement, aortic grafts, atherectomy, thrombectomy and thrombolysis.
  - Devascularisation by placement of embolising material as coils, particles and dedicated fluids.
  - Others such as ablation of varices, renal artery radiofrequency ablation (RFA), reperfusion by fibrinolysis, intravascular drug-therapy, TIPS and transjugular biopsy, IVC filter placement, valvuloplasty.
2. Non-vascular procedures
  - General, including puncture, drainage and evacuation. Placement of “assistance devices” such as fiducials.
  - Musculoskeletal: osteoplasty, kyphoplasty and placement of stabilisation materials, intervertebral disc treatment.
  - Abdominal organs (mainly liver and kidney): drainage, instillation therapies (e.g. in echinococcal cysts) and endoscopic procedures (e.g. endoluminal stenting).
  - Central nervous system: neuronavigation procedures (e.g. deep brain stimulation).

- ***IGT in tumours***

1. Trans-vascular procedures
  - Embolisation (bland, drug eluting, non-permanent)
  - Chemo-ablative (including organ-selective perfusion): chemo-perfusion and chemo-embolisation.
  - Radio-ablative
2. Non-vascular procedures
  - Thermally ablative, such as radiofrequency ablation, microwave ablation, cryo-ablation and laser ablation.
  - Thermo-mechanical ablative, as highly intensive focused US.
  - Electro-ablative, as irreversible electroporation.
  - Chemo-ablative, such as ethanol (acetoacid) injection.
3. IGT in supportive conditions
  - Pain therapy by instillation (drugs, alcohol and acetoacid, implant-like substances such as cement) or thermal coagulation.
  - Others, such as port-caths.
4. IGT in surgical procedures
  - Intraoperative fluoroscopy, US, CT and MRI guided, monitored and controlled procedures by either direct imaging of a target or probe placement.
  - Intraoperative angiography under C-arm fluoroscopy or DSA (plus flat panel CT-like techniques) and related interventions.
5. IGT in radiation oncology
  - Radiation treatment planning. Monitoring radiation therapy by navigator techniques and motions detection.
  - Fluoroscopic, US, CT or MRI guided placement of fiducials and radiation catheters (brachytherapy, after loading).

## Quality and medico-legal dependent characteristic of IGT

IGT involves a significant number of medical specialities. The involved procedures should have agreed technical, methodological and legal prerequisites before being constituted within an individual subspecialty. Defined prerequisites have to be fulfilled for equipment and materials, but also for medical environments to guarantee a safe and efficient practice.

Performing IGT necessitates specific quality management tools for establishing standards and maintaining levels of excellence. Therefore, radiologists should be involved in identifying and establishing centres of excellence for training and support (e.g. “help desk”), and establishing quality measures (e.g. definition of outcome parameters for specific procedures). Quality and safety standards have already been incorporated into IGT by several national and international organisations, scientific and professional societies (such as the Society of Interventional Radiology [SIR], Cardiovascular and Interventional Radiological Society of Europe [CIRSE], and European Society of Cardiology [ESC]).

A European task force group on IGT might be necessary to further develop certification guidelines and establish requirements for IGT practice according to known standards, focused on common recommendations and certification guidelines. The involved scientific and professional societies should collaborate in the recommendations on training programmes and accreditation of IGT practices, crossover knowledge and collaborative efforts. These documents will guide professionals in complementing the quality certification with high safety standards regarding clinical practice guidelines, appropriate equipment use, good operational techniques, proper training, and adequate medical environment. The proper definition of adequate targets and the use of structural, functional and molecular biomarkers in these procedures should be clearly defined and evaluated.

Moreover, within the European Organisation for Research and Treatment of Cancer (EORTC), an EORTC imaging group has already been created to ensure standardisation of image acquisition and quality assurance of imaging investigations (such as CT, PET-CT and MRI) used for EORTC trials. This group involves several subgroups, including a Radiology Technologies Committee and an Imaging in Radiotherapy Committee that essentially deals with IGT. The European Society of Radiology should lead in the production of a shared document with all the involved societies and organisations in this matter.

Training programmes organised by ESIR (European School of Interventional Radiology), certification by EBIR (European Board of Interventional Radiology), as well as numerous state-of-the-art documents on the performance and also radiation protection in IGT presented by CIRSE, are already well established and accepted initiatives, and should be promoted even more.

All IGT procedures involving ionising radiation should include the measurement and recording of appropriate technical equipment factors used in the procedure. These data should be standardised, displayed and recorded. The European Union Directive on Medical Exposures [3] considers the use of appropriate means of radiation protection and insists that special attention should be given to quality assurance programmes including quality control measures and patient dose assessment.

The European Commission has funded several research actions (such as DIMOND [4] and SENTINEL [5]) focused on radiation protection aspects such as recording radiation dose information. Development of local programmes implementing software-based models to collect and manage this information should be promoted.

Radiation protection is of utmost importance for both diagnostic and therapeutic procedures in IGT. The European Directive 97/43/EURATOM on medical exposure, article 9 [3], addresses interventional techniques, as an example of a procedure that can involve high doses of radiation to patients and establishes certain requirements for its practice. Most interventional radiology societies include radiation protection aspects as part of their quality programmes [6].

With respect to radiation protection, the United Nations Scientific Committee on the Effects of Atomic Radiation (UNSCEAR) [7], the International Commission on Radiological Protection (ICRP) [8] and the International Atomic Energy Agency (IAEA) have all contributed to improve radiation safety aspects in recent years.

## Strategies for increasing the “visibility” of IGT

Physicians within various medical specialties perform IGT. In some areas within this field, radiologists and radiology departments are not involved at all, while in others radiologists and other physicians have developed specific procedures. IGT performed by radiologists is almost always free from problems related to self-referral. From a medical point of view, this independent position should be recognised and brought to the attention of healthcare providers.

### Promotion of training and education in IGT

As image-guided and minimally invasive therapy progresses in complexity and volume with the development of new technical and biological knowledge, it becomes obvious that none of the existing medical specialities offer the ideal clinical and technological training on this field. Clinical, technical and biological fundaments are needed to adequately incorporate IGT procedures to the benefit of the patient.

In radiology, image guidance is the cornerstone of percutaneous and intravascular interventional procedures and assists in a targeted minimally invasive treatment approach. As a consequence, image guidance has to be an integral part of the residency training, incorporated into the curriculum of radiology and including all fluoroscopy, US, CT, and MR guidance techniques. Postgraduate education in IGT is also already provided by ESR and its subspecialty societies (such as CIRSE, ESGAR, ESUR and EUSOBI), the European School of Radiology (ESOR) and approved by a standardised examination (European Board of Interventional Radiology, EBIR). Nevertheless, to guarantee a comprehensive service in IGT, the promotion of postgraduate education on IGT has to be clearly extended.

For radiologists, a potential postgraduate education programme should include the acquisition of a recognised European board certification in interventional radiology, such as provided by the ESR and CIRSE (EBIR), or even a board certification in IGT. The European qualification is aimed at standardising comprehensive training and expertise in IGT and could be the first step in recognition of this sub-speciality.

If non-radiological specialities are incorporating the use of radiological techniques as IGT within their training curriculum or are planning a postgraduate educational activity involving imaging techniques, it seems mandatory to do this in concordance with the existing national and international requirements for education in radiation protection and patient safety, and in close cooperation with national radiological societies to maintain quality standards. Radiologists should be involved in these training programmes to guarantee these standards.

### Research issues and strategies

IGT research can be broadly divided into two categories, target specific research (e.g. the type of tumour or vascular lesion by imaging biomarkers) and technical research (e.g. evaluation of a new device or procedure). Understanding the efficacy and application of new and emerging technologies is a critical first step, which then leads to target-specific research. The focus of this research is aimed at understanding when, where and in whom the therapy can provide clear clinical benefit and how to use IGT in conjunction with, or as an alternative to, more established therapies. This also clearly includes research on the development and implementation of imaging biomarkers, defined as objectively measured indicators of normal biological processes, pathological changes, or responses to a therapeutic intervention [9].

Current IGT research has been hampered by lack of the type of funding provided by industry-led research such as is available for pharmaceutical, novel radiotherapy or surgical treatments. Construction of databases using pooled data from multiple centres, some of them financed by industry, is worthless unless there are very strict controls on data quality and organisation. Clinical specialists who lack the knowledge and expertise required to champion IGT and who are often already over-committed in pursuing their own research goals often dominate committees in control of other funding streams.

IGT research should focus on proof of concept and outcome using widely accepted clinical measures and imaging biomarkers of response. Whilst the cost effectiveness of IGT compared with conventional surgery (including follow-up imaging and allowing for recurrence) is usually fairly obvious, precise records will be needed to convince healthcare managers and government departments who are constantly searching for cheaper and less invasive treatments. ESR and the European Institute for Biomedical Imaging Research (EIBIR) can act as a catalyst for new research studies by linking academic and researching centres, co-ordinating protocols, facilitating data collection and analysis.

### Cooperation with other medical specialties/societies/clinical partners

The therapeutic role of IGT makes it part of the treatment algorithm in several diseases where this approach can be used as a combined, sequential or stand-alone treatment. In this regard, IGT providers must have extensive knowledge of the disease and processes they treat with specific focus on other therapeutic possibilities. Because IGT necessitates awareness, concern, questioning and improvement about therapeutic options for a given disease, a strong relationship with other medical specialties and medical societies treating the same type of disease by other means is mandatory, so that treatment options for a patient are standardised. This will allow improvement of knowledge by permanent discussion and exchange of information.

Moreover, validation of IGT obviously requires evaluation of the treatment outcomes that will inevitably require head-to-head comparison with other different medical/surgical/radiation therapies, and also evaluation of complex therapeutic strategies with the combination of IGT and other treatments. These evaluations will have to be performed with medical trials that can take place mainly through disease or organ-oriented societies (e.g. ESMO, ESTRO, ECCO, EORTC). This cooperation must involve IGT providers, other medical societies and medical specialist. Such collaboration by medical societies must reflect what takes place in daily practice with IGT providers investing time in multidisciplinary meetings to enable further dissemination of their technique and understanding of the disease treatment concepts.

### Non-professional public awareness

The visibility and acknowledgements for IGT amongst other medical specialties is still very low. The non-professional public awareness is even lower. Activities to increase awareness amongst non-professionals should aim for target groups, such as politicians, healthcare providers, local hospital administrators and patient’s organisations. All of these target groups play an important role in improving recognition, financing and support for IGT.

Politicians are probably the most difficult to influence, as they are usually not focused on a specific topic and have little commitment which reaches beyond their term of office. Lobby activities to probably get IGT on a political agenda will only work in relation to a major disease group, like diabetes and cancer, whereas the major issue of political interest is mainly the escalating cost of healthcare that necessitate comprehensive cost-effectiveness studies.

Healthcare providers, such as insurance companies, National Health Services and local hospital administrations are a more reliable and stable group with whom to enter discussion. However, they only have limited interests in cost-effectiveness, quality, and safety data. Activities in this area might be supported by cooperation with institutions such as NICE (National Institute of Clinical Excellence) in the UK.

As mentioned, patients play a very important role in promoting and supporting IGT. Creating more awareness among patients typically encompasses:

- Patient information provided by brochures and websites with up-to-date information on IGT.
- Promoting IGT in the news media and popular press.
- Promotion of direct access of patients to IGT (establishing contact via websites, IGT providers, hospital, national radiological societies).

## Health economics and health technology assessment

IGT can contribute to medical care in two main ways, and this will affect the calculations of cost effectiveness of the procedure, that is the cost per unit of patient benefit.

- Replacing or improving existing more invasive or less accurate techniques to deliver the same therapeutic result.
- Delivering a novel treatment, not otherwise available.

For those who fund healthcare, the best use of resources is achieved through promoting treatments that provide the greatest patient benefit at the lowest cost, thereby purchasing the maximum health gain for the population. This holds true not only for state-funded healthcare and insurance-based systems, but also for individually funded care. In deciding which techniques are the most cost effective, it is usually necessary to compare the health gain and the cost of a newer technique or procedure with the best conventional alternative approach if that method were not available. The degree of additional benefit in terms of cure or longer disease-free survival will be estimated from the available literature, and the costs of the procedure and treatment of any complications calculated.

IGT techniques often replace or modify a more invasive or less accurate procedure. If one assumes equal efficacy, then the best cost effective approach is the cheaper. Calculating cost is not always straightforward and should include all costs, including allowance for the total staff time, drug and equipment use, hospital bed usage, and the treatment of procedure-related complications. There are relatively few examples of published cost effectiveness analyses for IGT, many of these procedures being at least as effective as traditional techniques, with lower overall costs. Demonstration of the cost effectiveness of IGT methods of treatment and targeting with formal quantification of financial as well as patient benefit would encourage their wider adoption.

In a broad perspective, health technology assessment (HTA) might be the way for the systematic evaluation of health-relevant IGT procedures and methods, the effectiveness, safety and economic viability of a health intervention, as well as its social, ethical, legal and organisational effects; and for providing a basis for decisions in the health system.

## Conclusion

IGT has gained a substantial importance in healthcare services and should be considered a crucial component in the vast majority of therapy regimens that encompass nearly all medical fields. The major advantages of IGT are the preciseness and safety of the performed procedures based on imaging the adequate target. Due to the highly demanding technical requirements, specific training and skills, compliance to legal requirements is necessary in performing IGT.

Radiology, Nuclear Medicine, Surgery and Radiation Oncology are specialties that will benefit from this field. However, in terms of patient care therapy—and this may be especially true for IGT—imaging specialties will have to be deeply involved in the definition of the standards in IGT because agreements on the best procedures, parametric imaging results, measurements, analysis and presentations are essential in this rapidly evolving field. Radiologists should also take responsibility for the definition and compliance with the legal requirements regarding radiological imaging performed by non-radiologists.
